# Muscular Exercise

**Published:** 1881-03

**Authors:** W. W. Hall


### MUSCULAR EXERCISE.

BY DR. W. W. HALL.

EVERY bodily motion necessarily involves a loss of fluid and solid substances ; of fluids in insensible and visible perspiration amounting to a pound or more in twenty-four hours ; and of solid particles which are worn off by the friction of the muscles and other components of the system, as all friction implies waste and loss. A hard steel razor loses some of its substance, weighs less every time it is drawn along the strap ; otherwise it would not be sharpened by the operation.

If a knife is sharpened upon the edge of a table or bench, it will be very soon seen that the wood is wearing away ; all rubbing together wears off, detaches particles, and when it is remembered that in the human body there are several hundred muscles, and bands, and bones, moving on and across one another, and that the whole of the thirty or forty feet of the intestines are in incessant motion one against another ; that the heart is always throbbing; that the pulses in every part of the body are beating forever, it must be apparent that there is unceasing waste of solid particles, which no sooner are they detached than they are hurried along bodily towards the rectum, or the great receptacle of the solids of the body, or they are dissolved into the minutest particles and are conveyed by the fluids of the system into the bladder, and then passed outward in the urine, as seen in the sediment deposited as a solid substance on the bottom of the vessels containing it.

These losses occur day and night, by the incessant motion of parts over which we have no control. These are called involuntary motions, and wear off and out of the body a pound or more of its substance in a day, although we may remain in bed all the time ; but when, in addition to these, the voluntary actions of the muscles are put in operation, the hands and arms and feet and legs, with the various bends of the body, not less than two, three, or even half a dozen pounds may be passed out of the body in twenty-four hours, thus diminishing very largely the amount and bulk of the blood, lessening pain and inflammation, and returning the body to its healthful condition. Small wonder is it then that exercise, work, steady, unhurried labor, has such a decided, even powerful effect towards removing every variety of disease from the body, bringing back to it health and strength, and vigor and activity. Thus there are several distinct methods of curing disease, all efficient in their way, and more or less in repute by turns in the progress of the ages.

First. By direct bleeding, using the lancet or leeches.

Second. By the use of blisters, mustard plasters, irritants, or other modes of diverting the blood from diseased part.

Third. By the use of medicines which act on the bowels, or liver, or skin ; as by the employment of castor oil or salts for the bowels, calomel for the liver, and "sweats" for the skin, all these diminishing the amount of blood in the body.

Fourth. By ceasing to eat for a season, as food makes blood, and ceasing to eat cuts off the supplies, while the necessary operations of the system use up rapidly what is on hand. Hence, if we eat or drink nothing for two or three days, the weight of the body is diminished several pounds.

Fifth. By active exercise we work off and out of the body the waste, worn off, useless, and poisonous particles in the system to the extent of several pounds every day.

### AN APOSTROPHE.

Below her chin

There is a pin;

Above the pin

There is a chin:

Chin, pin,

Pin, chin;

Sweet chin,

Dear pin.

Below her nose

There is a rose;

Above the rose

There is a nose;

Nose, rose,

Rose, nose;

Sweet nose,

Dear rose.

"I am afraid the bed is not long enough for you," said the landlord to a seven-foot guest. "Never mind," he replied, "I'll add two more feet to it when I get in."

"Why, Franky !" exclaimed the mother at the summer boarding-house. "I never knew you to ask for a second piece of pie at home." "I knew 'twan't no use," replied Franky, quietly, as he proceeded with his pie-eating.

